# Where on the Differential Is Eisenmenger Syndrome in a Patient Without Prior Cardiopulmonary Disease?

**DOI:** 10.7759/cureus.8509

**Published:** 2020-06-08

**Authors:** Alec S Kellish, Abraham Hakim, Victoria Soal, Gabrielle Hassinger, Brian Gable

**Affiliations:** 1 Orthopaedics, Cooper Medical School of Rowan University, Camden, USA; 2 Psychiatry, Cooper Medical School of Rowan University, Camden, USA; 3 Internal Medicine, Cooper Medical School of Rowan University, Camden, USA; 4 Internal Medicine, Cooper University Hospital, Camden, USA; 5 Internal Medicine, Cooper Medical School of Rowan University-Cooper University Health Care, Camden, USA

**Keywords:** pulmonary hypertension, eisenmenger, congenital heart disease, atrial septal defect, pulmonary embolism, dyspnea, shortness of breath

## Abstract

Pulmonary hypertension (PH) can occur in patients with undiagnosed congenital heart disease, like atrial septal defects (ASDs), causing chronic left-to-right shunting. This may ultimately result in Eisenmenger physiology or syndrome (ES), a reversal of left-to-right shunting, resulting in a right-to-left shunt, thereby causing deoxygenated blood to enter systemic circulation as it bypasses the lungs. Development of PH due to an ASD is uncommon, and the occurrence of ES is <1% as most ASDs are corrected early in life. We present a 28-year-old female presenting with new-onset dyspnea found to have an undiagnosed ASD with ES.

A 28-year-old female without past medical history presented to the emergency department after a chest x-ray performed by her primary care physician (PCP) showed dilation of the pulmonary artery concerning PH. The patient reported a three-month history of progressively worsening intermittent palpitations and dyspnea, now unable to walk more than one block without becoming dyspneic. Further imaging studies revealed a 1.4 centimeters (cm) secundum ASD, 4.4 cm dilatation of the PA, a mean pulmonary artery pressure (PAPm) of 132 millimeters (mm) mercury (Hg), and Eisenmenger physiology. She was placed on pulmonary vasodilators and iron supplementation to address an underlying iron-deficiency anemia. The patient is stable on her current regimen and is undergoing evaluation for possible heart-lung transplantation at an outside hospital.

Dyspnea is one of the top 10 most common indications for emergency room visits annually. The differential diagnosis for dyspnea is vast, with ES, affecting only 0.8 in 1 million, far down on the list of possibilities, thus requiring high clinical suspicion to prompt further evaluation. Ultimately, the condition is preventable with early identification of underlying structural abnormalities for which definitive treatment options exist and are readily available, dramatically improving the prognosis if implemented before ES develops.

## Introduction

Shortness of breath, or “dyspnea”, is one of the most common presenting complaints of patients visiting the emergency department and comprises nearly one-quarter of patients evaluated in ambulatory clinics [1,2]. The differential diagnosis of dyspnea is expansive, ranging from common conditions including asthma and emphysema to the less common zebras, such as pulmonary hypertension (PH) and Eisenmenger syndrome (ES) [3].

PH is a term defined by a mean pulmonary artery pressure (PAPm) ≥ 25 millimeters (mm) mercury (Hg) while at rest [4]. This hemodynamic state is divided into five distinct sub-groups by the World Health Organization(WHO): pulmonary arterial hypertension (PAH), PH due to left heart disease, PH due to lung disease/hypoxia, chronic thromboembolic PH, and PH of multifactorial mechanisms [5]. The two most common etiologies of PH are left heart disease and pulmonary disease. If left uncorrected, certain types of congenital heart disease may also lead to sequelae of PH due to chronic left-to-right cardiac shunting, leading to right-sided cardiac overload and subsequent PAH, as seen in atrial septal defects (ASDs) [6]. The reversal of shunting, from "left-to-right” to “right-to-left” is pathognomonic for ES, occurring once the pressure in the right ventricle surpasses left ventricular pressure [7]. While congenital heart defects are often correctable, failure to correct these conditions may ultimately result in ES [8].

The development of PH due to an ASD is uncommon, while the development of ES is even less common, occurring in less than 1% of these patients [8,9]. The reversal of the shunt to a right-to-left shunt results in the systemic circulation of deoxygenated blood, as blood flow from the bypasses the lungs. Patients present with severe dyspnea on exertion, edema of the lower extremities, syncopal episodes, cyanosis, palpitations, lightheadedness, headaches, and changes in vision. Physically, patients may show signs of cyanosis, elevated jugular venous pressure, right ventricular heave, a palpable second heart sound, digital clubbing, hepatic congestion, ischemic ulcers, or livedo reticularis [7]. However, due to the chronic nature of the cardiopulmonary dysfunction and underlying hypoxia, subjective symptoms and physical examination findings may be unremarkable or absent, as the body can compensate for some degree of hypoxia [10]. As a result, patients may be undiagnosed for months or years due to physicians having an appropriately high threshold for proceeding with further diagnostic evaluation in the absence of clinical manifestations [11].

We present the case of a 28-year-old female presenting with three months of shortness of breath who was found to have an undiagnosed secundum ASD and PH.

## Case presentation

A 28-year-old Asian female presented to her primary care physician (PCP) for follow-up due to progressively worsening dyspnea on exertion. The patient reported that the dyspnea was increasingly impacting her ability to perform her day-to-day activities. Prior to the onset of this dyspnea, she was an active young adult who swam routinely, yet now found herself unable to ambulate one block or up on flight of stairs without severe dyspnea. Additionally, she endorsed occasional palpitations with the dyspneic episodes, though the palpitations never occurred independently of such episodes. She denied the presence of lightheadedness, syncope, chest pain, cough, sputum production, hemoptysis, paroxysmal nocturnal dyspnea, orthopnea, lower extremity edema, wheezing, fevers, chills, night sweats, or weight changes.

Notably, this 28-year-old female was a relatively new patient of her PCP. At the time of her initial PCP visit, she had endorsed mild dyspnea for which the differential included physical deconditioning secondary to a low back injury, anxiety, premature atrial contractions, asthma, and pulmonary embolism (PE). Her past medical history was remarkable only for a remote history of asthma when she was a child, and her use of oral contraceptives (OCPs). Her social history was remarkable for immigration to the United States from China 10 years ago. She was encouraged to keep a diary to track her symptoms and began physical therapy for a mechanical low back injury. Her physical examination at that time was unremarkable and she was able to tolerate all her physical therapy appointments, even hiking for five miles without difficulty five weeks prior to her admission. Of note, the patient did experience an episode of hemoptysis after a paroxysm of coughing while experiencing cold-like symptoms.

**Figure 1:**
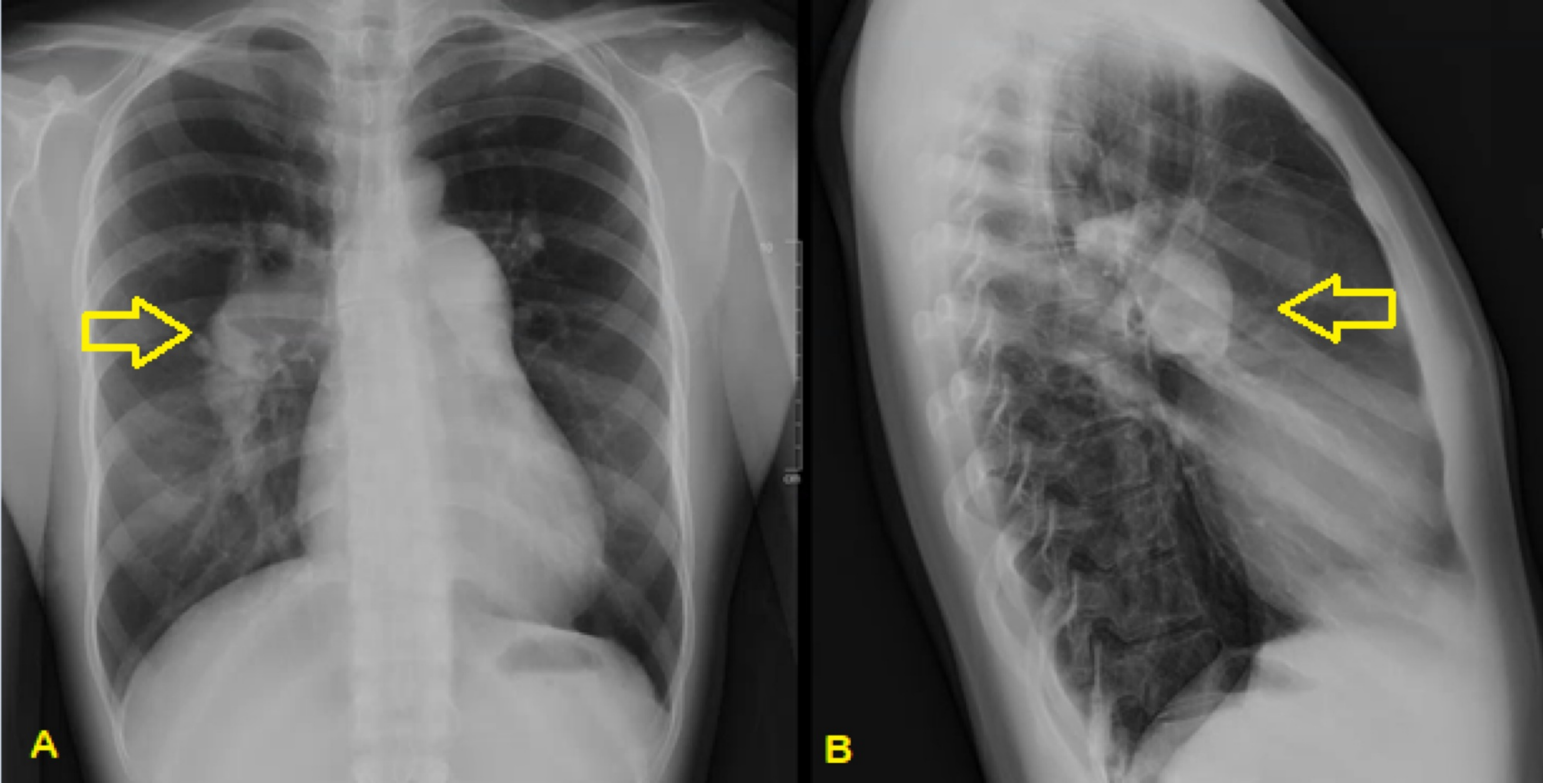
Anterior-posterior (A) and lateral (B) chest x-rays showing dilated pulmonary vasculature

On physical examination at her follow-up visit, she was noted to have visibly labored breathing, which resolved with rest and a normal cardiac exam, while her symptom diary showed progression in the severity of her symptoms. The differential diagnosis was expanded to include anemia in addition to the original differential. A chest x-ray was ordered, along with a complete blood count (CBC), D-dimer, and pulmonary function tests. The chest x-ray revealed prominent pulmonary vasculature concerning for PH (Figure 1). Subsequently, her PCP had the patient present immediately to the emergency department for further evaluation and intervention.

**Figure 2:**
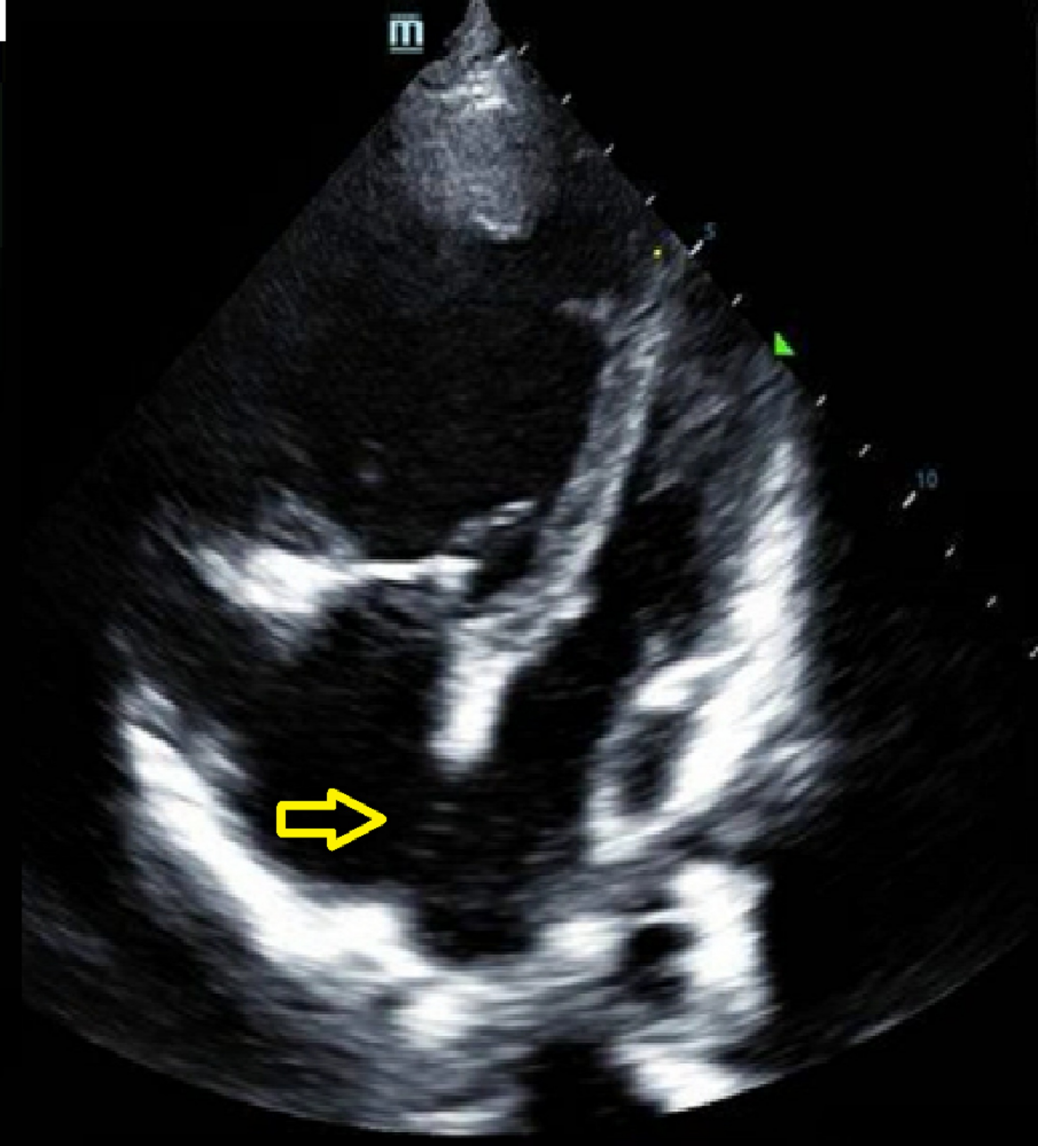
Apical four-chamber view transthoracic echocardiogram showing atrial secundum defect Arrow: atrial secundum defect

**Figure 3:**
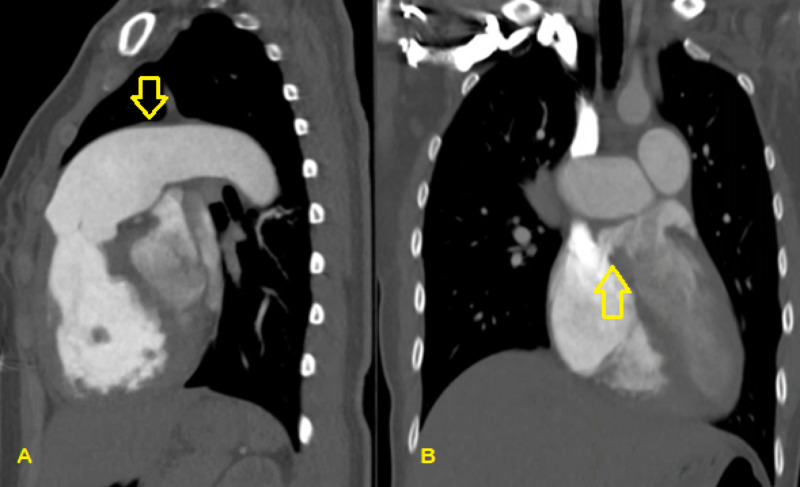
Sagittal (A) and coronal (B) views of a CT chest with contrast showing dilated pulmonary vasculature and atrial septal defect (A) Down arrow indicating 4.4 cm dilation of the main pulmonary artery; (B) up arrow indicating the location of the atrial septal defect

**Figure 4:**
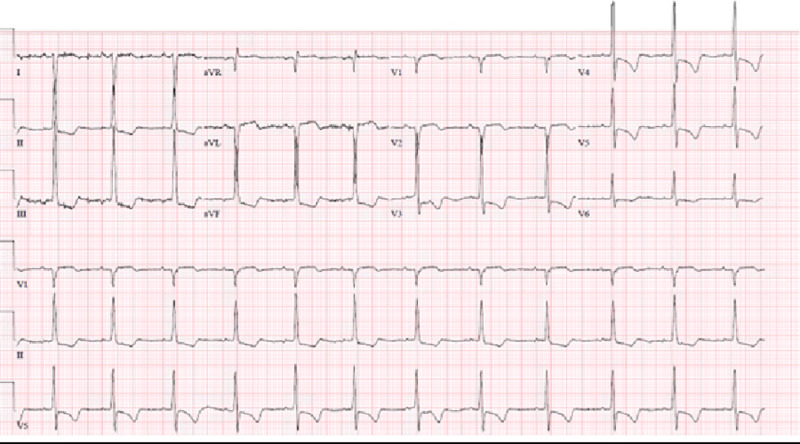
Electrocardiogram with evidence of right ventricular hypertrophy and inferior subendocardial ischemia

The patient was admitted after further diagnostic tests to evaluate the concerning chest x-ray showed an enlarged right ventricle and an ASD on bedside ultrasound, confirmed with a CT chest with contrast (Figure 2). On examination, there was a 3/6 systolic murmur present at the lower left sternal border, with no appreciable S1 or S2 splitting. Inspiratory and expiratory effort was normal with clear lung sounds, no signs of digital clubbing, no cyanosis, with the remainder of the exam completely benign. Her vital signs at rest included a blood pressure of 107/72 mmHg, heart rate 76 beats per minute, respiratory rate 18 breaths per minute, and oxygen saturation 92% on room air. During the interview and examination, the patient’s oxygen saturation was noted to fall to 72% without signs of dyspnea during the conversation, and she remained hypoxic despite 5L of oxygen via nasal cannula. The CT chest showed an ASD, 4.4 cm dilatation of the main pulmonary artery with dilatations of bilateral pulmonary arteries, segmental branches, and coronary sinus (Figure 3). Transthoracic echocardiogram revealed an ejection fraction of 50%, severe dilatation of the right atrium and ventricle, dilatation of the coronary sinus, a pulmonary artery pressure of 132 mmHg with right atrial pressure of 8 mmHg, moderate tricuspid regurgitation, and moderate pulmonic valve insufficiency. Her laboratory chemistries were unremarkable except for her iron studies showing a slight iron deficiency of 34 ug/dL (normal 42-135 ug/dL) and iron saturation of 8% (normal 15-50%) with her hemoglobin (12.6 g/dL) within normal limits. Her electrocardiogram showed normal sinus rhythm with right axis deviation, concern for septal infarction of undetermined age, and ST abnormalities suggesting inferior subendocardial injury (Figure 4).

 

She was transferred to a cardiac transplant center for further evaluation and potential heart-lung transplantation. Currently, she is stable on a medication regimen including ambrisentan and tadalafil. Her OCPs were discontinued. Since the initiation of pulmonary vasodilatory medications, she endorses improvement in her symptoms and is able to walk up to 10 blocks before becoming dyspneic. She is currently categorized as New York Heart Association Heart Failure Class 1.

## Discussion

Shortness of breath is a frequent, deceptively simple presenting symptom clinicians evaluate daily [3]. The differential diagnosis is incredibly lengthy and requires a thorough history and physical examination to determine if further diagnostic evaluation is indicated. However, even with a thorough patient history and physical examination, rare conditions like PH due to ES resulting from an undiagnosed ASD can be overlooked in favor of the more common diagnoses.

The differential diagnosis for dyspnea is built based on multiple factors, including temporal and situational nature, pathogenesis, and patient context [2]. In the case of the patient described above, the differential diagnosis for her dyspnea included physical deconditioning secondary to a low back injury, anxiety, premature atrial contractions, asthma, iron deficiency anemia, PE, and pulmonary PH once imaging was obtained. Physical deconditioning initially fit the temporal onset of the symptoms and situation onset, with the patient’s decrease in activity from a low back pain potentially lowering the patient’s cardiovascular fitness, thus resulting in dyspnea on exertion [12]. However, the dyspnea should have resolved with physical therapy and increased activity, but paradoxically worsened, making physical deconditioning less likely. Anxiety and psychogenic etiologies have been linked with dyspnea and have a plausible pathogenic mechanism, but did not fit with the onset of symptoms, or exacerbating factors. Furthermore, while the patient is a graduate student and reports moderate levels of stress, there had been no changes in her stress level for several years [13]. Asthma was high on the differential given the patient’s history of asthma and exacerbation of symptoms with exertion, in the context of physical activity being performed in the winter [2,3]. However, the development of asthma at 28-years-old after decades without symptoms is unlikely, and the patient did not endorse nor did physical exam find any wheezing. PE can also lead to dyspnea, and palpitations, similar to the symptoms our patient experienced [2,3,13]. However, outside of her OCP use, her history was not suggestive of a PE. While they can lead to acute onset of dyspnea that is exacerbated by increased activity, the dyspnea is not usually progressive over a two-month period. Additionally, while the patient was at a mild risk of developing a clot due to her OCP use, she had no other risk factors for thrombosis. Iron deficiency anemia is a less common, but significant cause of dyspnea, especially in female patients [3,13]. A lower than normal amount of hemoglobin decreases the availability of binding sites for oxygen and can compromise tissue oxygenation. This fits with the temporal onset and situational exacerbation of symptoms, as the body can reach a threshold where it is no longer able to compensate, and symptoms develop acutely. Furthermore, iron deficiency anemia can be associated with high-output heart failure, and cause palpitations, an associated symptom endorsed by our patient. Iron deficiency anemia fits well in the context of the patient, a young reproductive-aged thin female. During her evaluation, the patient was diagnosed with iron deficiency anemia in addition to her PH, ES, and ASD, and this was believed to be contributing to her hypoxia.

With an incidence of 0.8 cases per million adults, ES is far-down on the list of differentials, and even further down for patient’s without known cardiac disease, if even on the differential at all [7,14]. Congenital heart defects, like ASD, which can rarely lead to ES, are typically diagnosed at birth or during infancy, and rarely remain undetected until middle age in patients in receiving routine medical care [6]. In the evaluation of a patient with a remote history of childhood asthma presenting to his or her PCP with dyspnea and an unremarkable physical examination, extensive testing with suspicion for ES would be inappropriate if not unethical. This illustrates the importance of routine follow-up care, as these visits provide the clinician with more information as the symptoms progress, prompting changes in management and leading to an eventual diagnosis. Following the diagnosis of ES, the management of these patients is complex, requiring novel medical therapies, congenital defect repair, or even heart-lung transplantation. Unfortunately, treatment options for ES and PH are limited, with medication therapy often only providing modest improvements in symptoms, with survival rates of 77% at three years from the time of diagnosis with ES in patients without surgical intervention [15-17]. Traditional medical therapy involves calcium channel blockers, phosphodiesterase type 5 (PDE5) inhibitors, endothelin-1 antagonist, nitric oxide modulation, and prostacyclin agonists. The goal of these medications is to decrease pulmonary artery vasoconstriction via the promotion of smooth muscle relaxation and vasodilatory mechanisms [16,17]. Ultimately, definitive treatment of ES requires either a heart-lung transplant or a lung transplant with surgical repair of the underlying cardiac defect, such as an ASD. Reversal of the right-to-left shunt or eliminating the shunt with surgical repair loses viability as a treatment option as the pulmonary artery pressure rises [18]. The shunt serves as a “pressure-relief valve”, and closure would cause significant dysfunction and increased strain on the right heart muscle. Survival after transplantation shows moderate improvement versus non-transplant management, with a five-year survival of 51% and 10-year survival of 28% [19].

The evaluation and management of patients with new-onset dyspnea require clinicians to use their clinical judgment to determine the appropriate next steps in management. Physicians should evaluate these patients with “fresh eyes” and minimize their own biases based cursory review of a patient’s electronic medical record. In patients presenting with dyspnea in the absence of other symptoms, and an unremarkable past medical history, an initially conservative approach to patient management may be appropriate. Physicians should routinely follow with these patients, performing a thorough physical examination at each visit, while escalating the level of care when clinically appropriate, leading to the diagnosis of rare, but manageable conditions like ES.

## Conclusions

Dyspnea is a common chief complaint evaluated daily by physicians and accompanied by an extensive differential of possible etiologies. While dyspnea in young adults is typically caused by relatively benign pathology, it can be the first harbinger of a grim prognosis. ES is a devastating diagnosis most commonly diagnosed in young patients, carrying a bleak outlook due to a poor prognosis and lack of definitive therapeutic interventions. In western cultures, the diagnosis is rare but can occur as a result of an undiagnosed cardiac abnormality, such as an ASD. A high clinical suspicion is required to prompt a workup for possible PH and ES, as without abnormal physical exam findings, like a cardiac murmur or overt signs of hypoxia, other etiologies are more likely. Ultimately, the condition is preventable with early identification of underlying structural abnormalities for which definitive treatment options exist and are readily available.

## References

[1] Hutchinson A, Pickering A, Williams P, Bland JM, Johnson MJ (2017). Breathlessness and presentation to the emergency department: a survey and clinical record review. BMC Pulm Med.

[2] Berliner D, Schneider N, Welte T, Bauersachs J (2016). The differential diagnosis of dyspnea. Dtsch Arztebl Int.

[3] Niedermeyer J (2015). Dyspnea in airway and pulmonary diseases. Internist.

[4] Hoeper MM, Ghofrani HA, Grunig E, Klose H, Olschewski H, Rosenkranz S (2017). Pulmonary hypertension. Dtsch Arztebl Int.

[5] Prins KW, Thenappan T (2016). World Health Organization Group I pulmonary hypertension: epidemiology and pathophysiology. Cardiol Clin.

[6] Post MC (2013). Association between pulmonary hypertension and an atrial septal defect. Neth Heart J.

[7] Kaemmerer H, Mebus S, Schulze-Neick I (2010). The adult patient with Eisenmenger syndrome: a medical update after dana point part I: epidemiology, clinical aspects and diagnostic options. Curr Cardiol Rev.

[8] Therrien J, Rambihar S, Newman B, Siminovitch K, Langleben D, Webb G, Granton J (2006). Eisenmenger syndrome and atrial septal defect: nature or nurture?. Can J Cardiol.

[9] Wood P (1958). The Eisenmenger syndrome or pulmonary hypertension with reversed central shunt. Br Med J.

[10] Lai YC, Potoka KC, Champion HC, Mora AL, Gladwin MT (2014). Pulmonary arterial hypertension: the clinical syndrome. Circ Res.

[11] Vashisht A, Katakam N, Kausar S, Patel N, Stratton J (2015). Postnatal diagnosis of maternal congenital heart disease: missed opportunities. BMJ Case Rep.

[12] Mahler DA, Harver A, Lentine T, Scott JA, Beck K, Schwartzstein RM (1996). Descriptors of breathlessness in cardiorespiratory diseases. Am J Respir Crit Care Med.

[13] Morgan WC, Hodge HL (1998). Diagnostic evaluation of dyspnea. Am Fam Physician.

[14] Galie N, Manes A, Palazzini M (2008). Management of pulmonary arterial hypertension associated with congenital systemic-to-pulmonary shunts and Eisenmenger's syndrome. Drugs.

[15] Hopkins WE, Ochoa LL, Richardson GW, Trulock EP (1996). Comparison of the hemodynamics and survival of adults with severe primary pulmonary hypertension or Eisenmenger syndrome. J Heart Lung Transplant.

[16] McLaughlin VV, Sitbon O, Badesch DB (2005). Survival with first-line bosentan in patients with primary pulmonary hypertension. Eur Respir J.

[17] Humbert M, Sitbon O, Simonneau G (2004). Treatment of pulmonary arterial hypertension. N Engl J Med.

[18] Jain S, Dalvi B (2018). Atrial septal defect with pulmonary hypertension: when/how can we consider closure?. J Thorac Dis.

[19] Waddell TK, Bennett L, Kennedy R, Todd TR, Keshavjee SH (2002). Heart-lung or lung transplantation for Eisenmenger syndrome. J Heart Lung Transplant.

